# The Role of Cognitive Reserve in Coping with Subjective Cognitive Complaints: An Exploratory Study of People with Parkinson’s Disease (PwPD)

**DOI:** 10.3390/brainsci15080795

**Published:** 2025-07-25

**Authors:** Chiara Siri, Anna Carollo, Roberta Biundo, Maura Crepaldi, Luca Weis, Ioannis Ugo Isaias, Angelo Antonini, Maria Luisa Rusconi, Margherita Canesi

**Affiliations:** 1 U.O.C. Neurorehabilitation Unit, Parkinson’s Disease and Movement Disorders Center, Moriggia Pelascini Hospital, Via Moriggia Pelascini, 3, 22015 Gravedona ed Uniti, Italy; annacarollo30@gmail.com; 2 Parkinson Institute, ASST “G.Pini-CTO”, Via Emilio Bignami 1, 20126 Milan, Italy; ioannis.isaias@asst-pini-cto.it; 3 Department of General Psychology, University of Padua, Via Venezia, 8, 35131 Padua, Italy; roberta.biundo@unipd.it; 4 IRCCS, San Camillo Hospital, Via Alberoni, 70, 30126 Venice, Italy; luca.weis@unipd.it (L.W.); angelo.antonini@unipd.it (A.A.); 5 Department of Human and Social Sciences, University of Bergamo, Piazzale S. Agostino, 2, 24129 Bergamo, Italy; maura.crepaldi@unibg.it (M.C.); marialuisa.rusconi@unibg.it (M.L.R.); 6 Neurodegenerative Disease Unit, Centre for Rare Neurological Diseases (ERN-RND), Department of Neuroscience, Padua Neuroscience Center (PNC), University of Padua, Via Giustiniani, 5, 35128 Padua, Italy

**Keywords:** Parkinson disease, neurodegenerative disorder, subjective cognitive complaints, quality of life, cognitive reserve, cognition

## Abstract

Background/Objectives: Depression, anxiety and apathy are often associated with subjective cognitive complaints (SCCs) in people with Parkinson’s disease (PwPD) without cognitive impairment. Cognitive reserve (CR) enhances emotional resilience, allowing people to better cope with stress and emotional challenges, factors affecting quality of life. We aimed to explore the relationship between CR and mood/anxiety in cognitively intact PwPD with and without SCCs. Methods: In this cross-sectional study we enrolled 133 PwPD and normal cognitive function (age 59.8 ± 6.7 years; disease duration 9.0 ± 5.5 years; male/female 84/49). We assessed cognitive reserve (CR scale), subjective cognitive complaints (with PD-CFRS), QoL (PDQ8), mood, anxiety and apathy (BDI-II; STAI, PAS, Apathy scales). We used a *t*-test to compare groups (with/without SCC; M/F); correlations and moderation analysis to evaluate the relation between CR and behavioral features and the interplay between CR, behavioral discomfort and QoL. Results: The group with SCCs had significantly (*p* < 0.05) higher scores in PDQ8, Apathy, STAI, PAS-C and BDI-II scales than those with no SCCs. Males with SCCs had higher scores in PDQ8, Apathy scale and BDI-II while females differed in PDQ8 and Apathy scale scores. In the SCC group, late-life CR was negatively correlated with PAS-C (avoidance behavior) and BDI-II; correlations were confirmed in the male group where CR also correlated with PDQ-8 and PAS persistent anxiety. Conclusions: PwPD and SCCs are more depressed and anxious compared to people without SCCs. Furthermore, we found a relationship between depressive symptoms, anxiety and CR: PwPD with SCCs may rely on cognitive reserve to better cope with the feeling of anxiety and depression, especially in male gender.

## 1. Introduction

Parkinson’s disease (PD) is a neurodegenerative disorder characterized by motor and nonmotor symptoms. Rigidity, tremor, bradykinesia and instability are the main motor symptoms causing movement impairment; cognitive and behavioral alterations are common among the nonmotor symptoms. Cognitive alterations have been extensively studied: mild cognitive impairment (MCI) has been reported in up to 40% of patients, and dementia in 30–40% of patients, with the incidence increasing with disease duration and age, and MCI as a risk factor [1,2,3,4]. Behavioral and neuropsychiatric symptoms such as anxiety, depression, apathy and impulsive compulsive behaviors have been described and can be common (see [5] for a review): depression symptoms occur in up to 37% of the patients (17% meet criteria for major depression), significant anxiety symptoms were reported in about 25% of patients, mean apathy prevalence was 35% and impulse control disorders occurred in about 10–20% of the patients, with different prevalence rates for each disorder. Motor, neuropsychiatric and cognitive symptoms have been shown to impact the quality of life (QoL) [6,7].

The estimated prevalence of PD in high-Human-Development-Index European countries, such as Italy, is 3.58 cases per 1000, and projections predict a 3% increase in Italy by 2050 [8,9,10,11,12]. The growing prevalence and the disability associated with PD make it extremely important to better understand the nature of motor and nonmotor symptoms to help people with Parkinson’s disease (PwPD) and their caregivers cope with the deterioration in QoL [13].

Subjective cognitive complaints (SCCs) consist of the self-experienced difficulties in memory or other cognitive abilities despite achieving normal results at formal assessment. In PD, SCCs are two times more common than in the healthy population [14], may precede MCI [15] and can be present in up to 83% of PwPD with normal cognitive function [16]. Depression, anxiety and apathy have been associated with SCCs in PD [17], but the relationship between psychiatric disorders and SCCs is not clear yet.

Cognitive reserve (CR) is the brain’s ability to cope with brain damage using pre-existing cognitive procedures or enlisting compensatory processes [18]. Therefore, CR consists of individual differences in cerebral processes that allow some to cope better than others with brain pathology and to maintain brain functions.

Years of education have been used as a proxy to quantify CR, but a multidimensional conceptualization of the construct is more appropriate. Most common proxies’ indicators are education, occupation, physical and leisure activity, creativity and/or premorbid intelligence [19,20]. To date, many questionnaires have been developed to measure CR, and some have been used in PD, mainly to examine the relationship between CR and cognitive decline in PwPD [21,22,23].

Studies on CR in PD focused on its relationship with cognitive impairment and explored CR’s protective role, especially on executive functions. They confirmed that cognitive decline is delayed in PwPD who have higher CR, even if cognitive impairment progression is faster once started [24,25,26].However, to the best of our knowledge, no studies have examined whether CR has a role in mediating emotional responses to stressful situations, such as experiencing cognitive loss, albeit subjective, and deterioration in quality of life (QoL).

In this study, we want to evaluate whether CR modulates emotional responses in addition to preserving cognitive abilities. Therefore, this study aimed to explore the relationship between CR and the ability to cope with neuropsychiatric symptoms (i.e., anxiety, depression, apathy and impulsive compulsive behaviors) associated with SCCs in PwPD with normal cognition.

Furthermore, considering that mood and anxiety disorders are more common in the female gender in healthy populations [27,28,29,30], we wanted to explore eventual gender differences in how CR interacts with these psychiatric symptoms.

## 2. Materials and Methods

This cross-sectional study is part of a larger longitudinal and multicenter (Milano, Salerno and Venice) Italian study “Validation of Mild Cognitive Impairment criteria in Italian Parkinson’s disease patients” (GR-2016-02361986) which aims to explore PD’s cognitive and behavioral profile by improving cognitive and behavioral assessment instruments.

It includes 133 PwPD recruited in Milano (ASST-Azienda Socio Sanitaria Territoriale-Gaetano Pini-CTO) and Venezia (IRCCS, San Camillo Hospital) from August 2018 to December 2024.

Inclusion criteria were as follows: aged between 40 and 70 years; clinically confirmed diagnosis based on UK Brain Bank criteria (PD with at least 5 years of disease duration) and normal cognitive abilities as per cognitive assessment allowing for Level II criteria for PD MCI to be applied.

Exclusion criteria were as follows: linguistic comprehension deficit; an education level of less than 5 years; presence of severe sensory deficits; severe psychiatric, neurological and cardiovascular pathologies; and previous neurosurgical procedures.

The present study was approved by the Ethics Committees of the enrolment centers. Written informed consent was obtained from all study subjects after full explanation of the procedure involved. The research was completed in accordance with the Declaration of Helsinki [31].

### 2.1. Assessment

PD cognitive abilities were evaluated in the ON state by means of a comprehensive neuropsychological battery common to both recruitment sites, which allowed for the evaluation of PD-MCI as per Level II criteria [32]. Moreover, we assessed cognitive reserve (QCR [21]), SCCs (with PD-CFRS [33]), QoL (with PDQ8, [34]), mood, anxiety and apathy (by administering BDI-II, STAI scales, PAS scale and Apathy scale).

We collected data from the following scales/tests/questionnaires:

The Mini-Mental State Examination (MMSE) [35] and Montreal Cognitive Assessment (MoCA) [36] for global cognitive abilities.

The PD-Cognitive Functional Rating Scale (PD-CFRS) (Italian validation [33]): A brief questionnaire developed for PD which explores various functional aspects that may be affected by cognitive impairment. It contains 12 questions, each addressing different functional areas related to this cognitive decline.

Cognitive reserve questionnaire (QRC): To evaluate the cognitive reserve, we adapted the criteria for proxies proposed by Hindle and colleagues [24]; the scale evaluates early life (education), middle years (work activity) and late life (socialization/significant relationship).

Parkinson’s Disease Questionnaire PDQ-8 [34] was used to measure quality of life. This is a shortened version of the 39-item PDQ-39, a scale used to quantify the quality of life for individuals with Parkinson’s disease.

Parkinson Anxiety scale (PAS-A, PAS-B, PAS-C [37]) was used to evaluate anxiety symptoms. It includes three subscales evaluating persistent anxiety, episodic anxiety and avoidance behaviors and it has been specifically developed for the PD population.

The State–Trait Anxiety Inventory (STAI scales, trait or state) [38] is a 40-item self-report measure of anxiety using a 4-point Likert-type scale (from 0 to 3 points) for each item.

The Apathy scale [39] to measure apathy. This 14-item questionnaire has been proved to be a reliable and valid measure of apathy in PD.

Beck Depression Inventory-II (BDI-II) [40], a 21-item self-report inventory measuring the severity of depression in adolescents and adults and widely used in PwPD.

The Barratt Impulsiveness Scale (BIS-11) [41] was used to evaluate impulsivity. This is a 30-question self-report measure of impulsiveness which has been used in PwPD in many studies.

Questionnaire for Impulsive Compulsive Disorders in Parkinson’s Disease-Rating Scale (QUIP-RS) [42]: This questionnaire was used to evaluate behavioral addictions and compulsive behavior in PwPD and was used to quantify these symptoms.

### 2.2. Statistical Analysis

To explore the interplay between CR and SCCs in PwPD, we defined two subgroups based on the presence of subjective complaints. These subgroups are as follows: (a) PD-SCC, patients with a PD-CFRS score of 0 (no subjective complaint); and (b) PD-SCC+, patients with a PD-CFRS score of 1 or higher.

Categorical variables were reported as numbers and percentages, while continuous variables were reported as mean and Standard Deviation, or median and inter-quartile range, depending on their distribution (Gaussian or not), as assessed by the Shapiro–Wilk Test.

Between-group comparisons of demographic and clinical characteristics were conducted using the Chi-square test or the Fisher exact test for categorical and unpaired *t*-tests, or the Mann–Whitney U test for continuous variables, as appropriate; for all the analyses, *p*-values < 0.05 were considered to be statistically significant.

Differences between groups differentiated by the presence of SCCs overall and within gender-based groups were explored. Correlation analyses were applied using Spearman rank correlation to comprehensively evaluate the relationship between cognitive reserve and relevant behavioral characteristics with a comparison between males and females; we used moderation analysis to evaluate the extent to which cognitive reserve influences the symptoms and overall quality of life of patients with Parkinson’s disease and to explore eventual gender differences in how CR interacts with these psychiatric symptoms. We explore this possible modulation in the overall sample and with focus on the PD-SCC+ group.

We created dichotomous variables (Yes/No) for depression, general, state and trait anxiety, apathy and impulsivity, based on the cut-off scores. Additionally, we dichotomized the late life subscale of QCR (Low/High; cut-off median value =8) and assessed differences in percentages among groups and by gender with the Chi-square test. The analyses were conducted with Jamovi software version 2.3.8.0.

## 3. Results

Participants had a mean age of 60.3 years (±6.8) and a disease duration of 8.99 years (±5.5), with a distribution of 84 males and 49 females (see Table 1 for clinical and demographic characteristics).

### 3.1. Subjective Cognitive Complaints

Eighty-one PwPD reported SCCs (PD-CFRS ≥ 1; PD-SCC+ group) while 52 did not report SCCs (PD-CFRS = 0; SCC− group).

No differences were found when comparing the two groups in demographic and clinical data; the group PD-SCC+ had significantly higher scores in the PDQ8 t (117) (*p* < 0.001, d = 0.79), Apathy scale t (119) (*p* < 0.001, d = 0.71), STAI scales t (106) (*p* < 0.05, d = 0.42), PAS-C scale t (108) (*p* < 0.05, d = 0.42), BDI-II t (108) (*p* < 0.01, d = 0.48) and BIS11 t (105) (*p* < 0.01, d = 0.55) scale compared to the group SCC− (see Table 2). Only apathy and impulsivity were considered in terms of their presence/absence, and they resulted to be more frequent in PD-SCC+ at the Chi-square comparison.

We did not find any significant difference in clinical and demographic characteristics between people with higher CR late life apart from age, which was higher compared to PwPD and low CR late life (mean years: 61.2 vs. 58.7, *p* < 0.05).

### 3.2. Gender Differences

No differences emerge in male and females for behavioral characteristics and quality of life (Table S1). However, when separately considering gender, we observed that males with SCC+ had higher scores in the PDQ8 (*p* < 0.01), Apathy scale (*p* < 0.01) and BDI-II (*p* < 0.05) than SCC−, while females differed in the PDQ8 scores (*p* < 0.05) and Apathy (*p* < 0.01) scale but not in BDI-II scores (Tables S2 and S3). The difference in pathological scores was not significant as per the Chi-square test.

In males, we found significative negative correlations between CR late life and the apathy (r = −0.282, *p* < 0.05), QUIP RS (r = −0.448, *p* < 0.001), persistent anxiety (r = −0.261, *p* < 0.05) and avoidance behavior (r = −0.311, *p* < 0.010) subscales of PAS and PDQ8 (r = −0.229, *p* < 0.010); in females, we found CR late life to negatively correlate only with PAS-C (r = −0.394, *p* < 0.05).

### 3.3. Cognitive Reserve

CR (early-, middle- and late-life proxies) did not differ between the PD-SCC+ and PD-SCC− groups.

In the PD-SCC+ group, late-life CR was significantly correlated with PAS, avoidance behavior (r = −0.418, *p* < 0.001) and BDI-II (r = −0.248, *p* < 0.05); correlations that were confirmed and found to be stronger in the male group where late-life CR also correlated with the PDQ-8 (r = −0.475, *p* < 0.01) and persistent anxiety subtest of PAS (r = −0.348, *p* < 0.05).

Moderation analyses were conducted both in the all-sample and, in particular, in the PD-SCC+ group (Table 3; Tables S4 and S5).

**Figure 1:**
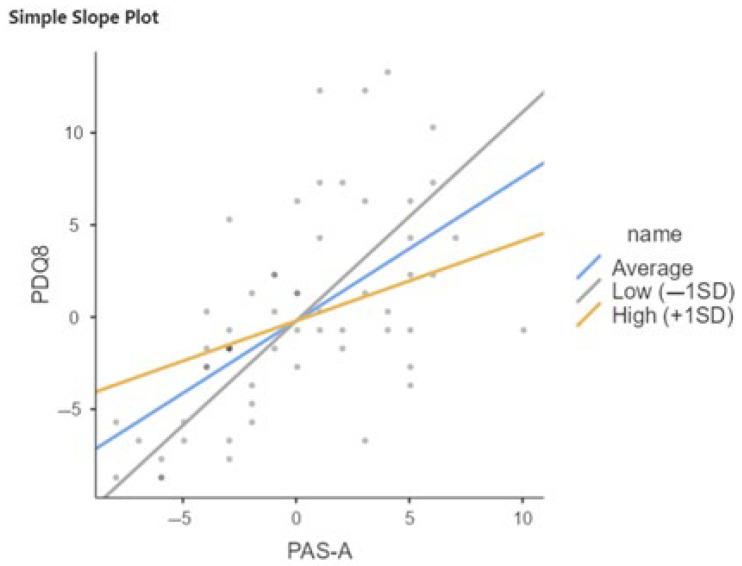
Relation between PAS-A and PDQ8 moderated by QRC total in total sample of PwPD. SD, Standard Deviation; Low = Average (Mean) QRC −1SD; High = Average (Mean) QRC +1SD.

Considering the total sample (PD-SCC+ and PD-SCC−), the interaction term between PAS-A and QRC late life is significant (b = −0.162, *p* < 0.05), suggesting that the effect of PAS-A on PDQ8 varies with late-life cognitive reserve (Table 3 and Table 4, and Figure 1).

**Figure 2:**
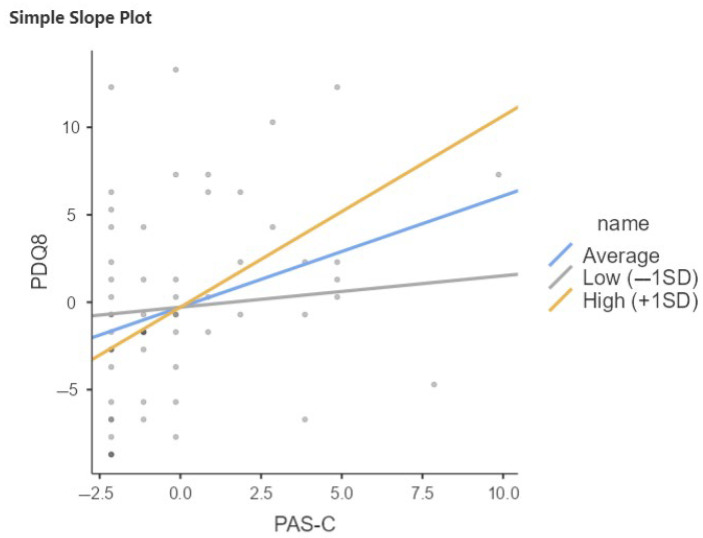
Relation between PAS-C and PDQ8 moderated by QRC total in total sample of PwPD. SD, Standard Deviation; Low = Average (mean) −1SD; High = Average (mean) +1SD.

Late-life cognitive reserve is also a moderator in the relationship between PAS-C and PDQ8; in particular, the interaction term is significant (b = 269, *p* < 0.001), indicating that the effect of PAS-C on PDQ8 increases with increasing late-life CR (Figure 2; Table 5).

**Figure 3:**
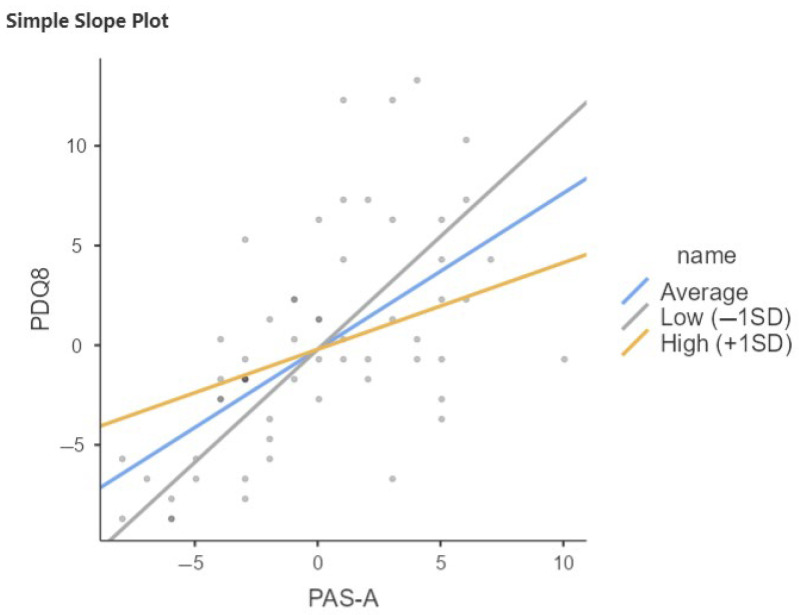
Relation between PAS-A and PDQ8 moderated by QRC late life in PD-SCC+. SD, Standard Deviation; Low = Average (mean) −1SD; High = Average (mean) +1SD.

The same results emerge in the PD-SCC+ group, where CR influences how anxiety is perceived in relation to scores on quality of life questionnaires. Notably, it is the late-life cognitive reserve—particularly concerning relationships with others—that shows significant scores in this relationship. In this group, the moderation analysis shows that the interaction term between PAS-A and QRC late life is significant (b = −0.212, *p* < 0.05 (Figure 3, Table 6)). Specifically, the association between anxiety and quality of life decreases with increasing late-life cognitive reserve. When late-life CR is low, the effect of PAS-A on PDQ8 is stronger; the higher the late-life CR, the weaker the effect. This moderation does not emerge in the SCC− group.

**Figure 4:**
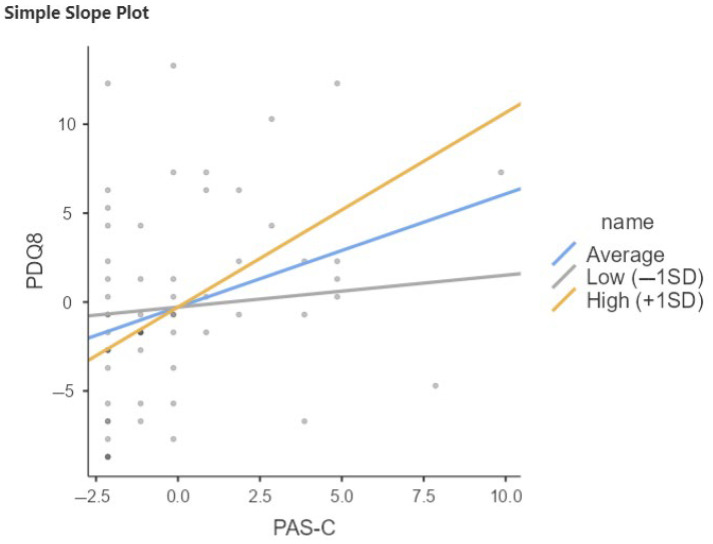
Relation between PAS-C and PDQ8 moderated by QRC late life in PD-SCC+. SD, Standard Deviation; Low = Average (mean) −1SD; High = Average (mean) +1SD.

The same trend emerges in the PD-SCC+ sample as in the total sample with respect to moderation of CR late life in the relationship between PAS-C and PDQ8 (Table 7, Figure 4). Notably, the interaction term is significant (b = 0.278, *p* < 0.001), indicating that again, the effect of PAS-C on PDQ8 increases with increasing late-life CR.

## 4. Discussion and Conclusions

Our study aimed to explore the effect of cognitive reserve on coping with the neuropsychiatric symptoms (i.e., anxiety, depression, apathy and impulsive compulsive behaviors) associated with SCCs in PwPD. We studied patients with unimpaired cognitive functions with and without cognitive complaints and explored the relationship between CR and neuropsychiatric symptoms in those populations.

In PwPD, both SCCs and CR have been studied regarding cognition [14,15,16,17,20,21,22,23,43,44,45,46]. However, this is the first study exploring how CR can help cope with neuropsychiatric symptoms and alterations in quality of life in PwPD with normal cognitive functions and SCCs.

Being an original topic, it is important to underline that it has been studied in a large and well-selected sample, since we included PwPD enrolled consecutively who were screened using a common cognitive assessment battery built to fit the Level II criteria for PD-MCI.

### 4.1. Subjective Cognitive Complaints

In agreement with the literature about the prevalence of SCCs in cognitively intact PwPD [17], about 60% of our sample reported SCCs. However, this rate is quite high compared to the median reported in the systematic review, and this may be due to the strict criterion we used to attribute a participant to the group without SCCs (PD-CFRS = 0), while other authors [44] used a different cut-off with the same instrument or applied different instruments [17,43].

Without a specific instrument or established criteria to evaluate SCCs, using the PD-CFRS allows for the detection of eventual subjective cognitive complaints. The 12 questions explore functional aspects sensitive to cognitive impairment, allowing the patient to better reflect on their abilities and to report eventual difficulties. Moreover, unlike other questionnaires, it minimizes the effect of motor bias when reporting subtle cognitive changes.

Furthermore, we did not find any difference between the two groups in demographic or clinical variables or general cognition as measured by MoCA and MMSE; those aspects did not even correlate with the PD-CFRS score, allowing for a better interpretation of the results. Indeed, a recent meta-analysis has shown that the relationship between poor performance in general cognitive tests and SCCs is weak [43]. Similarly, motor symptoms and dopamine replacement therapy were not found to be associated with SCCs.

We found that people with SCCs had higher scores in scales measuring quality of life, mood, anxiety, apathy and impulsivity, as expected considering previous studies [17,45,46]. We also found a higher prevalence of pathological scores in apathy and impulsivity in the PD-SCC+ group.

### 4.2. Gender Differences

When exploring the gender differences, we observed that males with SCCs have worse quality of life, are more apathetic and are more depressed than females with SCCs, while females differ in QoL and Apathy scale scores as well, but not in BDI-II scores. Considering the recent observation [47] of worse anxious–depressive symptoms with increasing age in women compared to men in a large population of 1509 PwPD, our finding may seem contradictory. However, it might be possible that from a “gender perspective”, intended as the role of norms, rules, stereotypes and cultural expectations based on biological gender, men could pay a higher toll than women when feeling to be less cognitively performant.

### 4.3. The Role of Cognitive Reserve

To our knowledge, this is the first study assessing the possible role of cognitive reserve in modulating strategies to cope with neuropsychiatric symptoms related to subjective cognitive complaints in Parkinson’s disease.

Cognitive reserve was considered separately in its three proxies (early, middle and late life), with early life considering education, middle life working experiences and late life social relationships. We found that in people experiencing SCCs, the late-life cognitive reserve proxy was the one most related to anxiety and depression, particularly in men, where it was correlated with quality of life too.

Interestingly, with the moderation analysis, we showed that late-life CR influences how anxiety is perceived in relation to scores on quality of life questionnaires, specifically in PD-SCC+. When quality of life is worse, persistent anxiety scores increase; however, this pattern is modulated by late-life CR, as the increase in anxiety is weaker in people with higher late-life CR. An opposite effect is noted when considering the effect of worse QoL on avoidance behavior, where people with higher late-life CR tend to have a greater increase in avoidance behavior. From a psychological point of view, this apparent contradiction may be explained as it is a way to cope with persistent anxiety arising from a poor quality of life: by using avoidance behaviors, people with higher CR can manage their anxiety. This may mean that, being more aware of their difficulties with social networking or social moments, patients may decide not to expose themselves to social situations unless they have a significant relationship with the attendees. Alternatively, they may rely more on phone video calls to maintain their relations.

Aside from this specific observation, our data show that late-life CR is an important factor correlating with apathy, anxiety and depressive symptoms in PwPD and subjective cognitive complaints. Frequent telephone calls and maintenance of relationships may be a feature of an active cognitive lifestyle, which is associated with a positive cognitive trajectory as people get older [6], and our data show it is related to better coping with SCCs and the neuropsychiatric symptoms associated with them.

Alongside cognitive deficits, neuropsychiatric symptoms have been shown to have a significant impact on QoL [48], and it is worth exploring ways to improve these aspects. While education and work life are scarcely modifiable in adult–elderly people, social interactions can be augmented, and this would lead to benefits for both patients and caregivers since it has been shown that higher perceived social contact is associated with a lower caregiver burden [49,50]. Furthermore, since we did not find any significant difference between people with high and low late-life CR apart from older age in the former, an intervention to increase social interactions is likely beneficial for all patients. Music, dance, yoga and other group activities have been shown to improve neuropsychiatric symptoms in these patients [51,52,53] and can be a way to implement sociality.

One limitation of the study is the definition of SCCs. Since there are no established criteria or scales to evaluate SCCs, we chose a criterion to define the presence of an SCC, which is reasonable but may have been too strict compared to the prevalence found in other samples. Generally, the lack of standardized measures for SCCs impedes comparing results. Furthermore, we considered only cognitively intact PwPD in our study, but cognitive complaints are present in MCI and PDD; therefore, our results cannot be generalized to the whole PD population.

In conclusion, our data confirmed the presence of higher neuropsychiatric symptoms in cognitively unimpaired PwPD and subjective cognitive complaints. They showed a relation with cognitive reserve being associated with social interactions in late life, especially in males. On one hand, we confirm previous findings showing higher anxiety, depression and apathy in PwPD and with SCCs; on the other hand, for the first time, the roles of cognitive reserve and social interaction in particular on these psychological and behavioral features are highlighted.

Further studies are needed to confirm our findings, explore the effect of other cognitive reserve proxies (such as premorbid intelligence, leisure activities and creativity) and evaluate programs that help patients and their caregivers implement social relationships.

## Figures and Tables

**Table 1:** Clinical and demographic characteristics of Parkinson’s patients with and without subjective cognitive complaints.

	*PD-SCC−* *N = 52*	*PD-SCC+* *N = 81*	df	*p*-Value
	Mean (SD)/ N(%)	Mean (SD)/ N(%)		
Gender (male)	25.6%	37.6%	131	0.673
Age at visit	59.06 (7.04)	61.06 (6.62)	131	0.099
Disease duration at visit	8.27 (5.28)	9.44 (5.60)	130	0.235
Age of onset	50.10 (7.92)	50.98 (8.83)	130	0.564
ADL	5.92 (0.27)	5.63 (0.71)	131	0.005
IADL	5.92 (1.40)	5.90 (1.49)	130	0.929
QRC early life	6.73 (2.11)	6.22 (2.59)	131	0.238
QRC middle life	6.25 (1.83)	6.19 (2.04)	131	0.853
QRC late life	8.79 (0.91)	8.56 (1.51)	129	0.323
QRC total score	21.77 (3.97)	20.87 (4.77)	129	0.264

**Table 2:** Significant differences in behavioral characteristics and Qol in PwPD with (PD-SCC+) and without (PD-SCC−) subjective cognitive complaints.

	PD-SCC−	PD-SCC+	df	*p*-Value
	Mean (SD)	Mean (SD)		
PDQ8	4.85 (4.32)	8.70 (5.21)	117	<0.001
APATHY	9.22 (4.04)	12.24 (4.39)	119	<0.001
STAY-II	33.91 (13.07)	39.14 (11.57)	106	0.030
STAY-I	30.93 (11.0)	34.5 (9.6)	107	0.079
PAS-A	6.96 (4.77)	7.95 (4.1)	108	0.244
PAS-B	1.89 (2.6)	2.8 (3.1)	108	0.107
PAS-C	1.11 (2.12)	2.14 (2.68)	108	0.032
BDI II	7.08 (5.9)	10.3 (7.1)	128	0.008
BIS11	45.20 (15.77)	53.76 (15.38)	105	0.006
QUIPRS	1.90 (5.13)	3.41 (6.44)	124	0.170

**Table 3:** QRC effect on behavioral symptoms and PDQ8 in PwPD in SCC+.

Behavior Scale	Moderator	Interaction Term	*p*-Value
PAS-A	QRC	PAS-A * QRC Total	0.093
	Early Score	PAS-A * Early Score	0.316
	Middle Score	PAS-A * Middle Score	0.180
	Late Score	PAS-A * Late Score	0.048
PAS-C	QRC	PAS-C * CR	0.090
	Early Score	PAS-C * Early Score	0.781
	Middle Score	PAS-C * Middle Score	0.140
	Late Score	PAS-C * Late Score	<0.001
BDI	CR	BDI-II * CR	0.131
	Early Score	BDI-II * Early Score	0.116
	Middle Score	BDI-II * Middle Score	0.649
	Late Score	BDI-II * Late Score	0.236
APATHY	CR	APATHY * CR	0.216
	Early Score	APATHY * Early Score	0.633
	Middle Score	APATHY * Middle Score	0.176
	Late Score	APATHY * Late Score	0.152
PAS-B	QRC	PAS-B * QRC Total	0.377
	Early Score	PAS-B * Early Score	0.177
	Middle Score	PAS-B * Middle Score	0.690
	Late Score	PAS-B * Late Score	0.780
QUIPRS	QRC	QUIPRS * QRC Total	0.091
	Early Score	QUIPRS * Early Score	0.067
	Middle Score	QUIPRS * Middle Score	0.306
	Late Score	QUIPRS * Late Score	0.580
STAY-I	QRC	STAIYI * QRC Total	0.740
	Early Score	STAIYI * Early Score	0.990
	Middle Score	STAIYI * Middle Score	0.832
	Late Score	STAIYI * Late Score	0.298
STAY-II	QRC	STAIYII * QRC Total	0.614
	Early Score	STAIYII * Early Score	0.863
	Middle Score	STAIYII * Middle Score	0.932
	Late Score	STAIYII * Late Score	0.266
BIS11	QRC	BIS11 * QRC Total	0.238
	Early Score	BIS11 * Early Score	0.108
	Middle Score	BIS11 * Middle Score	0.426
	Late Score	BIS11 * Late Score	0.722

“*” is the symbol for the interaction.

**Table 4:** Relation between PAS-A and PDQ8 moderated by QRC total in total sample of PwPD.

Simple Slope Estimates						
			95% Confidence Interval			
	Estimate	SE	Lower	Upper	Z	*p*
Average (Mean)	0.593	0.0991	0.3988	0.787	5.99	<0.001
Low (−1SD)	0.818	0.1521	0.5194	1.116	5.37	<0.001
High (+1SD)	0.368	0.1509	0.0726	0.664	2.44	0.015

Note: Shows the effect of the predictor (PAS-A) on the dependent variable (PDQ8) at different levels of the moderator (QRC late life).

**Table 5:** Relation between PAS-C and PDQ8 moderated by QRC total in total sample of PwPD.

Simple Slope Estimates						
			95% Confidence Interval			
	Estimate	SE	Lower	Upper	Z	*p*
Average (mean)	0.896	0.197	0.510	1.282	4.55	<0.001
Low (−1SD)	0.543	0.206	0.139	0.947	2.64	0.008
High (+1SD)	1.249	0.266	0.728	1.770	4.70	<0.001

Note: Shows the effect of the predictor (PAS-C) on the dependent variable (PDQ8) at different levels of the moderator (QRCl_total).

**Table 6:** Relation between PAS-A and PDQ8 moderated by QRC late life in PD-SCC+.

Simple Slope Estimates				
	Estimate	SE	Z	*p*
Average (mean)	0.772	0.136	5.69	< 0.001
Low (−1SD)	1.012	0.203	4.99	<0.001
High (+1SD)	0.533	0.193	2.76	0.006

Note: Shows the effect of the predictor (PAS-A) on the dependent variable (PDQ8) at different levels of the moderator (QRC_somma).

**Table 7:** Relation between PAS-C and PDQ8 moderated by QRC late life in PD-SCC+.

Simple Slope Estimates						
			95% Confidence Interval			
	Estimate	SE	Lower	Upper	Z	*p*
Average (mean)	0.637	0.258	0.131	1.143	2.466	0.014
Low (−1SD)	0.180	0.238	−0.287	0.647	0.755	0.450
High (+1SD)	1.094	0.333	0.441	1.746	3.285	0.001

Note: Shows the effect of the predictor (PAS-C) on the dependent variable (PDQ8) at different levels of the moderator (QRC late_life).

## Data Availability

The raw data supporting the conclusions of this article will be made available by the authors on request.

## Supplementary Materials

The following supporting information can be downloaded at: https://www.mdpi.com/article/10.3390/brainsci15080795/s1, Table S1: Comparison between males and females in behavioral characteristics and Qol in PwPD; Table S2: Comparison of behavioral characteristics and Qol between male PwPD with (PD-SCC+) and without (PD-SCC−) subjective complaints; Table S3: Comparison of behavioral characteristics and Qol between female PwPD with (PD-SCC+) and without (PD-SCC−) subjective cognitive complaints; Table S4: QRC effect on behavioral symptoms and PDQ8 in PwPD (total sample); Table S5: QRC effect on behavioral symptoms and PDQ8 in PwPD in SCC−.

## Abbreviations

The following abbreviations are used in this manuscript:

PD	Parkinson’s disease
PwPD	People with Parkinson’s disease
LEDD	Levodopa equivalent daily dose
DAED	Dopamine agonist equivalent daily dose
