# Supplementary material for: The Role of Cognitive Reserve in Coping with Subjective Cognitive Complaints: An Exploratory Study of People with Parkinson’s Disease (PwPD)
Source: Brain Sci. 2025 Jul 25;15(8):795. doi: 10.3390/brainsci15080795 (PMC12384381; doi:10.3390/brainsci15080795)
Supplement: Supplementary file 1 [file brainsci-15-00795-s001.zip › brainsci-3667418-supplementary.pdf]

Table S1 Comparison between male and female in behavioral characteristics and Qol in PwPD

	Male Mean (SD)	Female Mean (SD)	df	p value
**PDQ8**	7.00 (5.32)	7.20 (5.07)	117	0.937
**APHATY_total score**	11.19 (4.62)	10.67 (4.29)	119	0.554
**STAY1_total score**	32.39 (9.57)	34.10 (11.55)	107	0.407
**STAY2_total score**	35.87 (11.77)	38.90 (13.46)	106	0.226
**PAS-A**	2.06 (2.91)	3.05 (2.83)	108	0.085
**PAS-B**	6.69 (4.34)	9.03 (4.17)	108	0.007
**PAS-C**	1.47 (2.15)	2.13 (3.01)	108	0.231
**BDI-II**	8.27 (6.20)	10.33 (7.66)	128	0.096
**BIS11_total score**	49.89 (14.16)	50.92 (19.30)	105	0.775

Table S2 Comparison in behavioral characteristics and Qol in PwPD between male with (PD-SCC+) and without (PD-SCC-) subjective complaints

	SCC+ Mean (SD)	SCC- Mean (SD)	df	p value
**PDQ8**	5.19 (4.30)	8.60 (5.60)	72	0.006
**APHATY_total score**	9.52(4.12)	12.50 (4.61)	72	0.005
**STAY1_total score**	30.55 (9.08)	33.85 (9.82)	68	0.154
**STAY2_total score**	33.71 (11.61)	37.63 (11.76)	67	0.170
**PAS-A**	1.61 (2.69)	2.41 (3.06)	68	0.258
**PAS-B**	6.29 (4.87)	7.00 (3.90)	68	0.500
**PAS-C**	1.13 (2.17)	1.74 (2.12)	68	0.238
**BDIII**	6.55 (4.99)	9.43 (6.70)	80	0.038
**BIS11_total score**	46.61 (13.30)	52. 49 (14.45)	68	0.085

Table S3 Comparison in behavioral characteristics and Qol in PwPD between females with (PD-SCC+) and without (PD-SCC-) subjective

	SCC+ Mean (SD)	SCC- Mean (SD)	df	p value
**PDQ8**	4.19 (4.43)	8.86 (4.67)	43	0.002
**APHATY_total score**	8.65 (3.94)	11.86 (4.10)	44	0.013
**STAY1_total score**	31.79 (14.83)	35.40 (9.33)	37	0.355
**STAY2_total score**	34.36 (16.33)	41.44 (11.12)	37	0.116
**PAS-A**	2.47 (2.39)	3.40 (3.06)	38	0.319
**PAS-B**	8.33 (4.40)	9.44 (4.06)	38	0.424
**PAS-C**	1.07 (2.09)	2.76 (3.32)	38	0.055
**BDIII**	8.06 (7.31)	11.70 (7.65)	46	0.111
**BIS11_total score**	41.85 (20.79)	55.83 (16.90)	35	0.033

Table S4 QRC effect on Behavioral symptoms and PDQ8 in PwPD (total sample)

Behavior Scale	Moderator	Interaction Term	P-Value
**PAS-B**	CR	PAS-B*CR	0.234
	Early Score	PAS-B * Early Score	0.735
	Middle Score	PAS-B * Middle Score	0.172
	Late Score	PAS-B * Late Score	**0.048**
**PAS-C**	CR	PAS-C*CR	**0.007**
	Early Score	PAS-C * Early Score	0.285
	Middle Score	PAS-C * Middle Score	**0.041**
	Late Score	PAS-C * Late Score	**<.001**
**BDI**	CR	BDI*CR	0.059
	Early Score	BDI * Early Score	0.128
	Middle Score	BDI * Middle Score	0.154
	Late Score	BDI * Late Score	0.160
**APATHY**	CR	APATHY*CR	0.324

Early Score	APATHY * Early Score	0.938
Middle Score	APATHY * Middle Score	0.073
Late Score	APATHY * Late Score	0.328

Table S5 QRC effect on Behavioral symptoms and PDQ8 in PwPD in SCC-

Behavior Scale	Moderator	Interaction Term	P-Value
**PAS-B**	CR	PAS-B*CR	0.507
	Early Score	PAS-B * Early Score	0.939
	Middle Score	PAS-B * Middle Score	0.133
	Late Score	PAS-B * Late Score	0.335
**PAS-C**	CR	PAS-C*CR	**0.011**
	Early Score	PAS-C * Early Score	**0.025**
	Middle Score	PAS-C * Middle Score	0.074
	Late Score	PAS-C * Late Score	0.116
**BDI**	CR	BDI*CR	0.081
	Early Score	BDI * Early Score	0.185
	Middle Score	BDI * Middle Score	0.070
	Late Score	BDI * Late Score	0.160
**APATHY**	CR	APATHY*CR	0.371
	Early Score	APATHY * Early Score	0.747
	Middle Score	APATHY * Middle Score	0.675
	Late Score	APATHY * Late Score	0.448
